# Over-Expression of Platelet-Derived Growth Factor-D Promotes Tumor Growth and Invasion in Endometrial Cancer

**DOI:** 10.3390/ijms15034780

**Published:** 2014-03-18

**Authors:** Yuan Wang, Haifeng Qiu, Weixu Hu, Shaoru Li, Jinjin Yu

**Affiliations:** 1 Department of Obstetrics and Gynecology, the Affiliated Hospital of Jiangnan University and the Fourth People’s Hospital of Wuxi, Wuxi 214062, Jiangsu, China; E-Mail: wxsydrf@hotmail.com; 2 Department of Obstetrics and Gynecology, International Peace Maternity & Child Health Hospital of the China Welfare Institute Affiliated to Shanghai Jiaotong University, Shanghai 200030, China; E-Mail: haifengqiu120@gmail.com; 3 Department of Radiation Oncology, Zhongshan Hospital of Fudan University, Shanghai 200032, China; E-Mail: haifengqiu120@163.com; 4 Department of Obstetrics and Gynecology, the First Affiliated Hospital of Xinxiang Medical University, Xinxiang 453100, Henan, China; E-Mail: binzhouliang@gmail.com

**Keywords:** endometrial cancer, platelet-derived growth factor-D (PDGF-D), proliferation, invasion, matrix metalloproteinase (MMP), the epithelial-mesenchymal-transition (EMT)

## Abstract

The platelet-derived growth factor-D (PDGF-D) was demonstrated to be able to promote tumor growth and invasion in human malignancies. However, little is known about its roles in endometrial cancer. In the present study, we investigated the expression and functions of PDGF-D in human endometrial cancer. Alterations of PDGF-D mRNA and protein were determined by real time PCR, western blot and immunohistochemical staining. Up-regulation of PDGF-D was achieved by stably transfecting the pcDNA3-PDGF-D plasmids into ECC-1 cells; and knockdown of *PDGF-D* was achieved by transient transfection with siRNA-PDGF-D into Ishikawa cells. The MTT assay, colony formation assay and Transwell assay were used to detect the effects of PDGF-D on cellular proliferation and invasion. The xenograft assay was used to investigate the functions of PDGF-D *in vivo.* Compared to normal endometrium, more than 50% cancer samples showed over-expression of PDGF-D (*p* < 0.001), and high level of PDGF-D was correlated with late stage (*p* = 0.003), deep myometrium invasion (*p* < 0.001) and lympha vascular space invasion (*p* = 0.006). *In vitro*, over-expressing PDGF-D in ECC-1 cells significantly accelerated tumor growth and promoted cellular invasion by increasing the level of MMP2 and MMP9; while silencing PDGF-D in Ishikawa cells impaired cell proliferation and inhibited the invasion, through suppressing the expression of MMP2 and MMP9. Moreover, we also demonstrated that over-expressed PDGF-D could induce EMT and knockdown of PDGF-D blocked the EMT transition. Consistently, in xenografts assay, PDGF-D over-expression significantly promoted tumor growth and tumor weights. We demonstrated that PDGF-D was commonly over-expressed in endometrial cancer, which was associated with late stage deep myometrium invasion and lympha vascular space invasion. Both *in vitro* and *in vivo* experiments showed PDGF-D could promote tumor growth and invasion through up-regulating MMP2/9 and inducing EMT. Thus, we propose targeting PDGF-D to be a potent strategy for endometrial cancer treatment.

## 1. Introduction

During recent decades, the incidence of endometrial cancer has been increasing in most regions of the world [1,2]. There will be about 49,500 new cases and 8200 deaths in the United States in 2013 [3]. Despite the more than 70% cases were diagnosed at early stage, as much as 28% of patients’ occurred regional or distant metastasis. Unfortunately, their prognosis were usually pretty poor (a 5-year survival rate of <40%) [4]. However, up to date, our knowledge about the initiation and progression of endometrial cancer is still limited. Recently, several studies demonstrated that platelet-derived growth factor-D (PDGF-D) play key roles in tumor growth and invasion, which might shed light on the investigations about endometrial cancer [5,6].

PDGF-D belongs to the PDGFs family [7–9], which was proved to be involved in various cellular procedures, including proliferation, migration, invasion, transformation, and survival, both in development and during pathogenesis [10,11]. As previously reported, PDGF-D mainly interacts with PDGFR-β (PDGF receptor-β) to activate its downstream signaling pathways, containing wnt/β-catenin, PI3K/Akt/mTOR and NF-κB, which finally accelerated the tumorigenesis and progression [6,12]. In human beings, over-expression of PDGF-D was found in pancreatic carcinoma, gastric carcinoma, renal carcinoma and ovarian cancer, implying the close relationships between high PDGF-D levels and human malignant tumors [13–16].

As little is known about PDGF-D in human endometrial cancer, herein, we aim to investigate its expression in endometrial cancer tissues, and further explore its functions during tumorigenesis and progression of endometrial cancer.

## 2. Results

### 2.1. Frequent Over-Expression of PDGF-D in Human Endometrial Cancer

**Figure 1.**
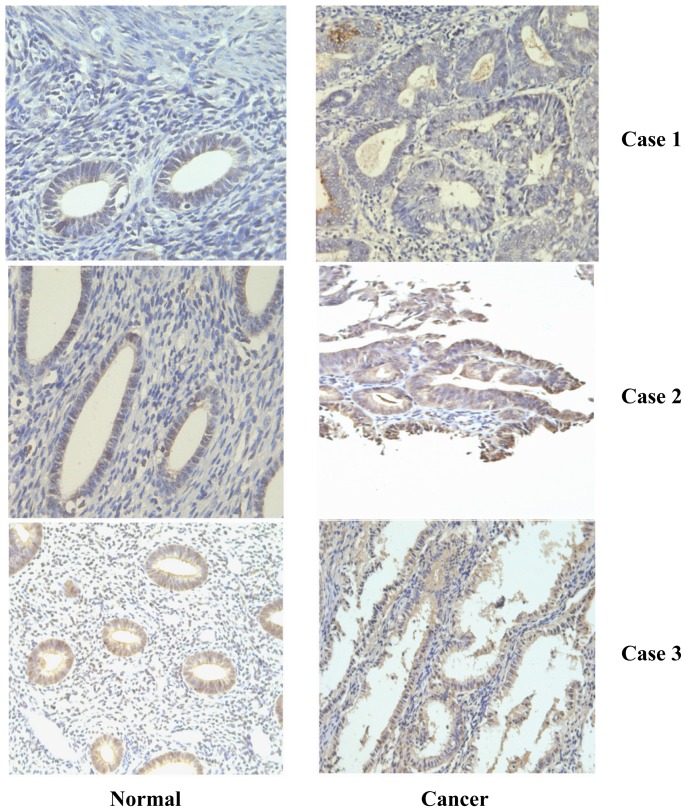
The protein expression in endometrial cancer and their corresponding normal endometrium tissues. Case 1: (endometrioid adenocarcinoma, grade III): platelet-derived growth factor-D (PDGF-D) was over-expressed in the cancer tissue; Case 2: (uterus serous cancer): PDGF-D was over-expressed in the cancer tissue; Case 3: (endometrioid adenocarcinoma, grade III): no significant up-regulation of PDGF-D was detected in the cancer tissue.

**Figure 2.**
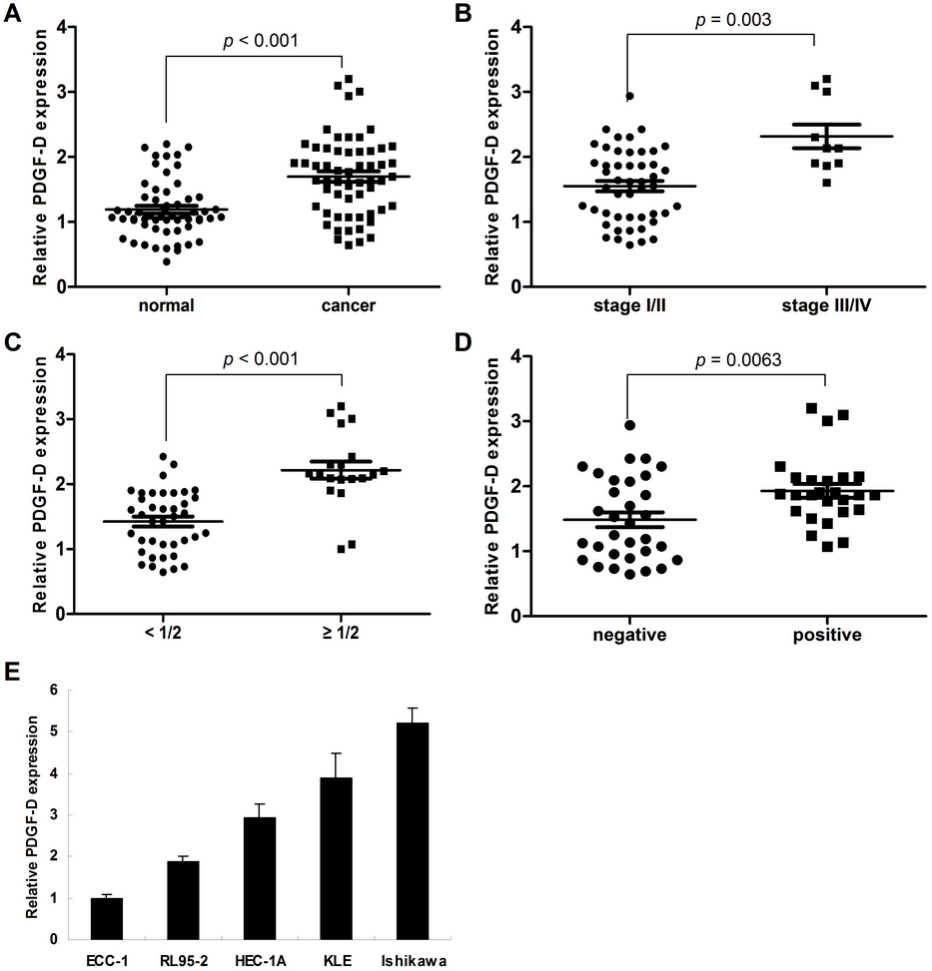
The relationship between PDGF-D over-expression and clinico-pathology characteristics. (**A**) The protein of PDGF-D was notably higher in endometrial cancer than those in the normal tissues (*p* < 0.001); (**B**) PDGF-D over-expression was associated with late stage (stage III/IV, *p* = 0.003); (**C**) PDGF-D over-expression was associated with deep myometrial invasion (*p* < 0.001); (**D**) PDGF-D over-expression was associated with lympha vascular space invasion (*p* = 0.006); (**E**) Among the five endometrial cancer cell lines, ECC-1 has the lowest and Ishikawa has the highest mRNA expression of PDGF-D.

Compared to the their adjacent normal tissues, 32 of 58 (55.2%, Table 1) endometrial cancer samples presented the over-expression of PDGF-D (*p* < 0.001, Figures 1 and 2A); moreover, we found that high level of PDGF-D was related to late tumor stage (*p* = 0.003, Figure 2B), deep myometrium invasion (*p* < 0.001, Figure 2C) and lympha vascular space invasion (*p* = 0.0063, Figure 2D). No relationship was detected between PDGF-D expression and patient’s age, histology grade and lympha node metastasis.

Among the five endometrial cancer cell lines, ECC-1 has the lowest and Ishikawa has the highest mRNA expression of PDGF-D (Figure 2E).

### 2.2. Up-Regulation of PDGF-D Accelerated Cell Proliferation and Invasion in ECC-1 Cells

**Figure 3.**
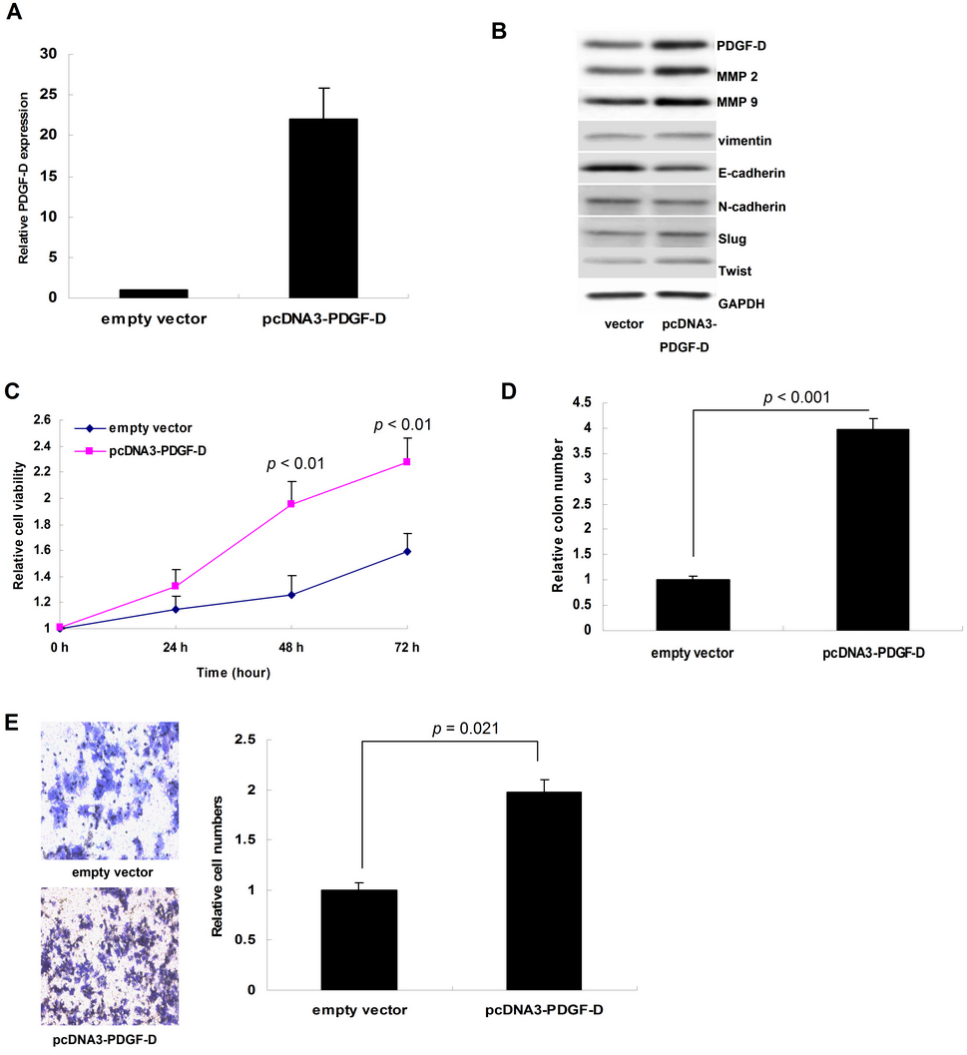
In ECC-1 cells, up-regulation of PDGF-D accelerated cell proliferation and invasion through increasing matrix metalloproteinase (MMP)2, MMP9 and promoting epithelial-mesenchymal-transition (EMT). (**A**) PDGF-D mRNA was up-regulated after transfection with pcDNA3-PDGF-D; (**B**) PDGF-D protein was up-regulated after transfection, and MMP2, MMP9, vimentin, Slug and Twist were increased, while E-cadherin was decreased; (**C**) PDGF-D over-expression promoted cellular growth in MTT assay (*p* = 0.028); (**D**) PDGF-D over-expression enhanced the colony formation (*p* = 0.012); (**E**) PDGF-D also notably enhanced the cellular invasion in Transwell assay (*p* < 0.001).

After transfection with pcDNA3-PDGF-D plasmid, both the mRNA and protein of PDGF-D were up-regulated in ECC-1 cells (Figure 3A,B). Abundant PDGF-D promoted cellular growth and colony formation significantly (*p* = 0.028 for MTT assay and 0.012 for colony formation assay, Figure 3C,D). Moreover, PDGF-D also notably enhanced the invasion of ECC-1 cells (*p* < 0.001, Figure 3E); as shown by western blot, the proteins of MMP2, MMP9, vimentin, Slug and Twist were up-regulated by PDGF-D, while E-cadherin was decreased (Figure 3B).

### 2.3. Silencing PDGF-D Suppressed Tumor Growth and Invasion in Ishikawa Cells

**Figure 4.**
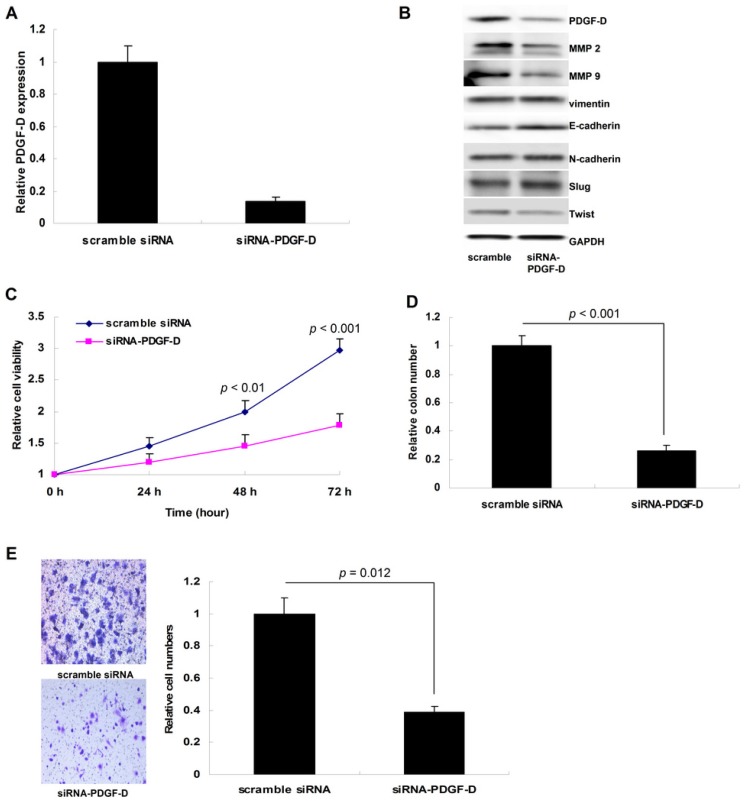
In Ishikawa cells, down-regulation of PDGF-D impaired cellular proliferation and inhibited tumor invasion through reducing MMP2 and MMP9, and suppressing EMT. (**A**) PDGF-D mRNA was decreased after siRNA-PDGF-D treatment; (**B**) PDGF-D protein was also decreased, the expression of MMP2, MMP9 and Twist was simultaneously inhibited, while E-cadherin level was notably up-regulated; (**C**) Loss of PDGF-D suppressed cellular growth significantly (*p* = 0.031); (**D**) Loss of PDGF-D suppressed cellular colony formation (*p* = 0.009); (**E**) Down-regulation of PDGF-D impaired the invasion of Ishikawa cells in Tranwell assay (*p* = 0.02).

By transient siRNA interference, we down-regulated PDGF-D in Ishikawa cells (Figure 4A,B). Loss of PDGF-D suppressed cellular growth and colony formation significantly (*p* = 0.031 for MTT assay and *p* = 0.009 for colony formation assay, Figure 4C,D). In addition, down-regulation of PDGF-D notably impaired the invasion of Ishikawa cells in the Tranwell assay (*p* = 0.02, Figure 4E); similarly, loss of PDGF-D decreased the level of MMP2, MMP9 and Twist, while increased E-cadherin expression (Figure 4B).

### 2.4. PDGF-D Promoted Tumor Xenografts Growth

**Figure 5.**
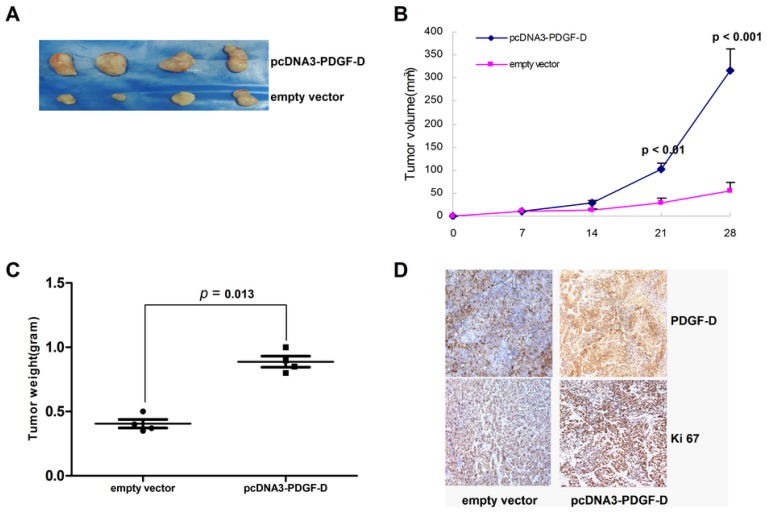
In nude mice, PDGF-D promoted the growth of ECC-1 xenografts. (**A**) The general picture of tumors formed by ECC-1 cells; (**B**) Compared to the negative control, over-expression of PDGF-D significantly promoted the growth of ECC-1 xenografts (*N* = 4 in each group, *p* < 0.01 on the 21th day and *p* < 0.001 on the 28th day); (**C**) The tumor weights were also increased by PDGF-D notably (*p* = 0.013); (**D**) In the tumor tissues with high PDGF-D, the Ki 67 protein was significantly up-regulated.

As shown in Figure 5A,B, elevated PDGF-D significantly accelerated the growth of ECC-1 xenogfrafts (*N* = 4 in each group, *p* < 0.01 on the 21st day and *p* < 0.001 on the 28th day). Consistently, the tumor weights were also notably increased by PDGF-D (*p* < 0.013, Figure 5C). Using immunochemical staining, we confirmed up-regulation of both PDGF-D and Ki 67 proteins in the ECC-1 xenografts (Figure 5D).

### 2.5. High PDGF-D Copy Number could Predict Worse Disease-Free Survival

**Figure 6.**
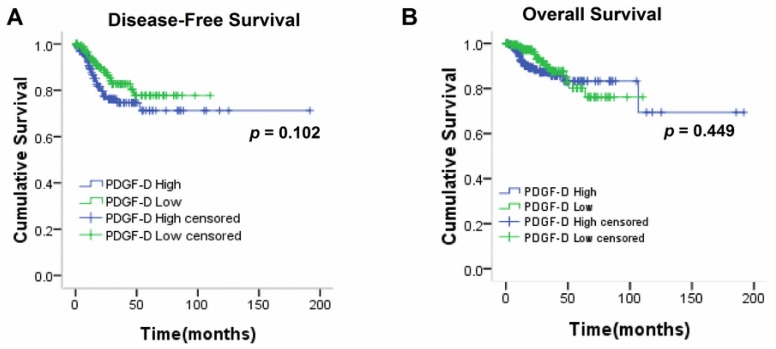
High PDGF-D copy number could predict worse disease-free survival. (**A**) High PDGF-D copy number could predict shorter disease-free survival in endometrial cancer patients (*p* = 0.102); (**B**) PDGF-D copy number did not affect the overall survival of endometrial cancer patients (*p* = 0.449).

By screening and analyzing the TCGA database, we found that high PDGF-D copy number might be a marker to predict disease-free survival in endometrial cancer patients (*p* = 0.102, Figure 6A), but not the overall survival (*p* = 0.449, Figure 6B).

## 3. Discussion

Endometrial cancer is the most common malignancy of the female genital system, which is often associated with excessive estrogen exposure, obesity and other risk factors [1]. In China, the incidence of endometrial cancer has been increasing and shifting to the younger population [17,18]. However, up to date, mechanisms underlying the initiation and progression of endometrial cancer remain elusive, further exploration on this disease is urgently required.

Several genes like *PDGF-D* were proved to be involved in human malignancies. Over-expression of PDGF-D was detected in pancreatic cancer, prostate cancer, gastric cancer, breast cancer and ovarian cancer, but rare in normal tissues, indicating the potential roles of PDGF-D in tumorigenesis and development [6,19]. In the present study, we found frequent over-expression of PDGF-D in more than 50% human endometrial cancer tissues, and elevated PDGF-D was related to late tumor stage, deep myometrium invasion and lympha vascular space invasion, highlighting the important roles of PDGF-D in the growth and invasion of endometrial cancer.

Previous studies had demonstrated that overabundant PDGF-D enhanced cell proliferation, migration and invasion was present in human cancer cells, and several signaling pathways were found to be involved [5,20,21]. In pancreatic cancer, by activating the Notch and NF-κB signaling pathways, PDGF-D enhanced tumor growth, promoted cellular invasion and decreased apoptosis [15]; and over-expression of PDGF-D also led to higher invasiveness in prostate cancer cells, through up-regulating the mTOR pathway (via targeting S6K and 4E-BP1) and down-regulating phosphorylation of Akt [22]. The study about human renal cell carcinoma showed that elevated PDGF-D induced angiogenesis and metastasis in a mouse model, in which the expression of angiopoietin-1 and MMP9 were increased [13]. In breast cancer, PDGF-D could activate CXCR4 to promote lymphatic metastasis [23].

According to our results, over-expressing PDGF-D in ECC-1 cells significantly accelerated tumor growth and promoted cellular invasion via increasing the level of MMP2 and MMP9 (which could impair the basilar membrane); while silencing PDGF-D in Ishikawa cells impaired cell proliferation and inhibited the invasion, through suppressing the expression of MMP2 and MMP9.

We also detected that PDGF-D promoted the processes of EMT in endometrial cancer cells. Morphologically, EMT is the processes by which epithelial cells lost the apical-basal polarity and acquired mesenchymal characteristics such as increased invasive and motility characteristics [24]. The most frequently used marker to identify epithelial cells from mesenchymal cells was E-cadherin (an epithelial marker), which is lost upon EMT and could lead to migration. The mesenchymal markers such as vimentin, Snail, Slug, ZEB1 and Twist were also commonly used to detect EMT [25]. Recently, several studies suggested that PDGF-D plays a critical role in the processes of EMT, which can enhance cellular migration and invasion [26,27]. In the present study, we also detected that PDGF-D could suppress E-cadherin and increase Slug and Twist expression, indicating the roles of PDGF-D in the migration and invasion of endometrial cancer.

Further, we investigated whether PDGF-D level could affect the survival of endometrial cancer patients. By analyzing the data from TCGA database, we found that patients with high PDGF-D copy number were prone to have worse disease-free survival, though the *p* value was not <0.05. Intriguingly, PDGF-D copy number did not affect the overall survival in endometrial cancer, which is probably caused by the mixture data containing various pathologic subtypes and unadjusted parameters like patients’ age or some other unknown factors. These findings need to be validated in our future studies.

## 4. Experimental Section

### 4.1. Tissue Samples Collection

Fifty-eight endometrial cancer tissues and their adjacent normal endometrium tissues were collected from the Fourth People’s Hospital of Wuxi (Wuxi, Jiangsu, China). All patients provided consent and approval was obtained from the ethics committee. All the patients were diagnosed following the criteria of the International Federation of Obstetrics and Gynecology (FIGO 2009).

### 4.2. Immunohistochemistry

The immunohistochemical staining was performed strictly as described previously [19]. The primary antibody against human PDGF-D was purchased from Invitrogen (Carlsbad, CA, USA). The slides incubated without anti-PDGF-D were used as the negative control. For evaluation of PDGF-D’s protein level, classification standards were as follows: negative (<10% tumor cells positively stained) and positive (≥10% tumors cells positively stained).

### 4.3. Cancer Cell Lines and Culture

Five endometrial cancer cell lines were used in this study: Ishikawa, KLE, RL95-2, ECC-1 and HEC-1A. All the cells were routinely cultured in the appropriate cultured medium (DMEM/F12 for KLE and RL95-2, RPMI1640 for Ishikawa and ECC-1, and Mcoy’s 5A for HEC-1A) (Gibco, Carlsbad, CA, USA). All the medium was supplemented with 10% FBS and the cell were cultured at 37 °C, 5% CO_2_.

### 4.4. Over-Expressing PDGF-D in ECC-1 Cells

Briefly, the plasmid pcDNA3-PDGF-D was constructed as previously described [19]. Then the mixture of Lipofectamine 2000 (Invitrogen, Carlsbad, CA, USA) and pcDNA3-PDGF-D or the empty vector was transfected into ECC-1 cells for 48 h. G418 (Sigma, St. Louis, MO, USA) was used to select the clones of ECC-1 cells stably over-expressing PDGF-D. qRT-PCR and western blot were performed to confirm the up-regulation of PDGF-D.

### 4.5. Knockdown of PDGF-D Using siRNA in Ishikawa Cells

Specific siRNA targeting human PDGF-D were purchased from Genepharmacy Inc. (Shanghai, China) [20]. Transfection of siRNA-PDGF-D or the scramble siRNA was performed using Lipofectamine 2000. Forty-eight hours after transfection, Ishikawa cells were harvest for qRT-PCR and western blot to confirm the knockdown of PDGF-D.

### 4.6. MTT Assay

Cellular proliferation was determined by MTT assay. Briefly, 4 × 10^3^ ECC-1 or 2 × 10^3^ Ishikawa cells/well were seeded into the 96-well plates and incubated overnight, then the cells were transfected with pcDNA3-PDGF-D or siRNA-PDGF-D. Seventy-two hours later, 5 μL MTT solution (5 mg/mL; Sigma, St. Louis, MO, USA) was added into each well and incubated for 1 h at 37 °C, then the formazan crystal was dissolved in 100 μL of DMSO (Sigma, St. Louis, MO, USA). The absorbance was measured on a plate-reader at 570 nm. All procedures were repeated in triplicate.

### 4.7. Colony Formation Assay

Two-hundred cells/per well were plated into six-well plates and transfected with siRNA-PDGF-D or pcDNA3-PDGF-D. These cells treated with scramble siRNA or empty vectors were set as negative control. Cells were routinely cultured for two weeks. Then the colony (containing more than 50 cells) number was counted under a microscope. This procedure was repeated in triplicate.

### 4.8. Cell Invasion Assay

Transwell chamber system (Millipore, Billerica, MA, USA) was used to investigate the alterations of cellular invasion. In these assays, the upper champers were pre-coated with 50 μL Matrigel at a 1:4 dilution (BD Biosciences, San Jose, CA, USA), and incubated at 37 °C for 2 h. Briefly, 1 × 10^5^ ECC-1 or Ishikawa cells were pre-transfected with pcDNA3-PDGF-D or siRNA-PDGF-D for 48 h, then the equal amount of cells were put onto the upper chamber and 500 μL condition medium was added into the bottom chamber. After 24 h incubation, cells on the bottom membrane were fixed, stained and counted under an inverted microscope. The empty plasmids and scramble siRNA were used as the negative control. The experiments were performed in triplicate.

### 4.9. RNA Extraction and qRT-PCR Amplification

Total RNA was extracted with the Trizol (Invitrogen, Carlsbad, CA, USA) according to the manufacturer’s instructions. The qRT-PCR assay was performed on ABI 7500 (Applied Biosystems, San Jose, CA, USA). The primers and conditions used in this study were same as previously studies [20]. GAPDH was used as the endogenous control. All the experiments were performed in triplicate.

### 4.10. Western Blot

Total protein was extracted using RIPA buffer (Beyotime, Shanghai, China). Equal amount of protein (30–50 μg) was separated on the 12% SDS-PAGE, transferred to PVDF membrane, and incubated with anti-PDGF-D (Invitrogen, Carlsbad, CA, USA), MMP2 and MMP9 (Santa Cruz, Dallas, TX, USA). The antibodies for EMT associated protein (including vimentin, E-cadherin, N-cadherin, Slug and Twist) were all obtained from Cell Signaling (Danvers, MA, USA). Following incubation with the secondary antibody, the bands of specific protein on the membranes were developed with enhanced chemiluminescence (Beyotime, Shanghai, China). GAPDH (Boster Technology Company, Wuhan, China) was used as the endogenous control.

### 4.11. Tumor Xenograft Assay

All the mice were handled according to the approval of Jiangnan University Animal Care and Use Committee. Five to Six weeks old female nude mice (Balb/c, obtained from Slac Laboratory Animal Co. Ltd., Shanghai, China) were used for this assay. To establish the endometrial cancer model, 1 × 10^6^ ECC-1 cells transfected with pcDNA3-PDGF-D or empty vector were injected subcutaneously into the right flank. Tumor diameters were recorded once a week using the calipers. Tumor volume = (length × width^2^)/2. Twenty-eight days after injection, all the mice were sacrificed to obtain the tumor for further analysis.

### 4.12. TCGA (The Cancer Genome Atlas) Data Extraction and Analysis

We obtained the survival data from TCGA website and reanalyzed. The patients were divided into two groups (the cut-off value = mean PDGF-D copy number): PDGF-D high group and PDGF-D low group. Kaplan-Meier curve was used to compare the differences between the two groups on disease-free survival and overall survival.

### 4.13. Statistical Analysis

SPSS 17.0 (SPSS Inc., Chicago, IL, USA) was used for the statistical analysis. χ^2^ test and *t*-test were used appropriately for different categories of data. *p* < 0.05 was defined to be statistically significant.

## 5. Conclusions

In summary, we found that PDGF-D was commonly up-regulated in human endometrial cancer, which was related with late stage, deep myometrial invasion and lympha vascular space invasion. *In vitro* and *in vivo*, we proved that PDGF-D significantly promoted tumor growth and invasion through up-regulating MMP2 and MMP9, and inducing EMT. Moreover, we also detected that high PDGF-D copy number might predict the worse disease-free survival. Based on these findings, we propose that targeting PDGF-D is a potent strategy for endometrial cancer treatment.

## Supplementary Information



## Figures and Tables

**Table 1.** Summarization of patients’ clinico-pathological characteristics.

Variables	Cases(n)	%
**Age**		

<55	25	43.1%
≥55	33	56.9%

**Stage**		

I	38	65.5%
II	10	20.7%
III	9	12.1%
IV	1	1.7%

**Grade**		

I	36	62.1%
II	12	20.7%
III	10	17.2%

**Myometrial invasion**		

<1/2	39	67.2%
≥1/2	19	32.8%

**Lympha node metastasis**		

Negative	18	64.3%
Positive	10	35.7%

**Lympha vascular space invasion**		

Negative	32	55.2%
Positive	26	44.8%
