# Supplementary material for: Over-Expression of Platelet-Derived Growth Factor-D Promotes Tumor Growth and Invasion in Endometrial Cancer
Source: Int J Mol Sci. 2014 Mar 18;15(3):4780–94. doi: 10.3390/ijms15034780 (PMC3975424; doi:10.3390/ijms15034780)
Supplement: Supplementary file 1 [file ijms-15-04780-s001.pdf]

Supplementary Information

Figure S1. (**a**) Mesenchymal morphology of ECC-1 cells induced by PDGF-D over-expression (200×); (**b**) Epithelial morphology of Ishikawa cells induced by knockdown of *PDGF-D* (200×).

(**a**)

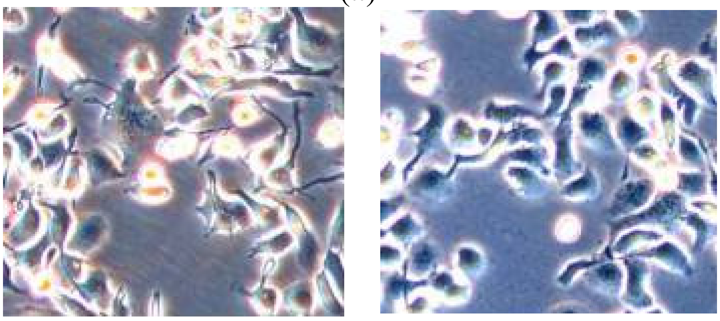

Empty vector pcDNA3-PDGF-D

(**b**)

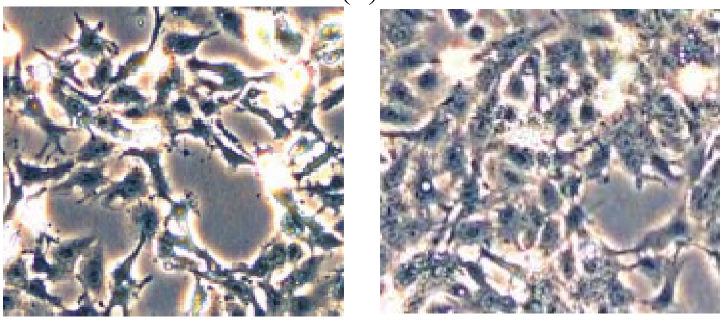

Scramble siRNA siRNA-PDGF-D

Figure S2. (**a**) The densitometric analysis and statistical analysis for Figure 3B; (**b**) the densitometric analysis and statistical analysis for Figure 4B.

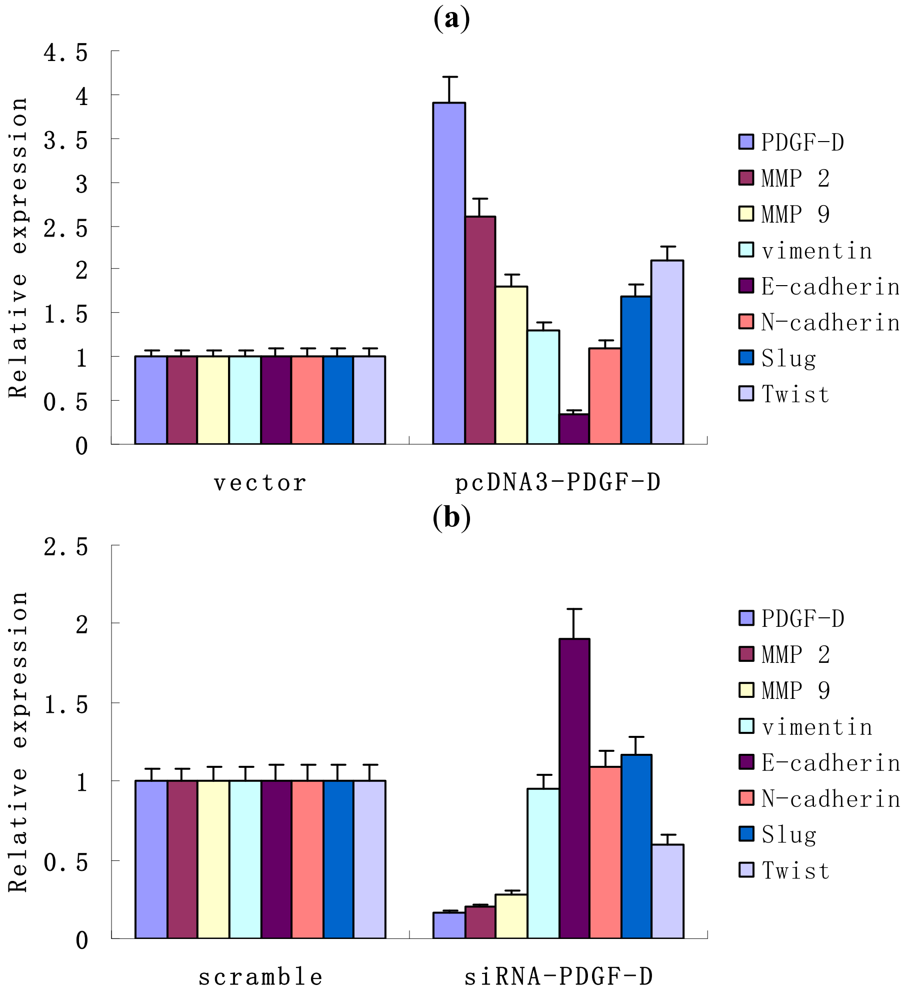

© 2014 by the authors; licensee MDPI, Basel, Switzerland. This article is an open access article distributed under the terms and conditions of the Creative Commons Attribution license (http://creativecommons.org/licenses/by/3.0/).
